# TREM2 gene induces differentiation of induced pluripotent stem cells into dopaminergic neurons and promotes neuronal repair via TGF‐β activation in 6‐OHDA‐lesioned mouse model of Parkinson's disease

**DOI:** 10.1111/cns.14630

**Published:** 2024-02-13

**Authors:** Hanbai Liang, Ping Liu, Zijing Wang, Huan Xiong, Cheng Yin, Dongdong Zhao, Chunhui Wu, Longyi Chen

**Affiliations:** ^1^ Department of Neurosurgery, Sichuan Provincial People's Hospital, School of Medicine University of Electronic Science and Technology of China Chengdu China; ^2^ Department of Gastroenterology and Hepatology, West China Hospital Sichuan University Chengdu China; ^3^ School of Life Science and Technology University of Electronic Science and Technology of China Chengdu China

**Keywords:** dopaminergic neurons, induced pluripotent stem cells, neuronal repair, Parkinson's disease, TGF‐β pathway, TREM2

## Abstract

**Objective:**

Induced pluripotent stem cells (iPSCs) hold a promising potential for rescuing dopaminergic neurons in therapy for Parkinson's disease (PD). This study clarifies a TREM2‐dependent mechanism explaining the function of iPSC differentiation in neuronal repair of PD.

**Methods:**

PD‐related differentially expressed genes were screened by bioinformatics analyses and their expression was verified using RT‐qPCR in nigral tissues of 6‐OHDA‐lesioned mice. Following ectopic expression and depletion experiments in iPSCs, cell differentiation into dopaminergic neurons as well as the expression of dopaminergic neuronal markers TH and DAT was measured. Stereotaxic injection of 6‐OHDA was used to develop a mouse model of PD, which was injected with iPSC suspension overexpressing TREM2 to verify the effect of TREM2 on neuronal repair.

**Results:**

TREM2 was poorly expressed in the nigral tissues of 6‐OHDA‐lesioned mice. In the presence of TREM2 overexpression, the iPSCs showed increased expression of dopaminergic neuronal markers TH and DAT, which facilitated the differentiation of iPSCs into dopaminergic neurons. Mechanistic investigations indicated that TREM2 activated the TGF‐β pathway and induced iPSC differentiation into dopaminergic neurons. In vivo data showed that iPSCs overexpressing TREM2 enhanced neuronal repair in 6‐OHDA‐lesioned mice.

**Conclusion:**

This work identifies a mechanistic insight for TREM2‐mediated TGF‐β activation in the regulation of neuronal repair in PD and suggests novel strategies for neurodegenerative disorders.

## Graphical abstract

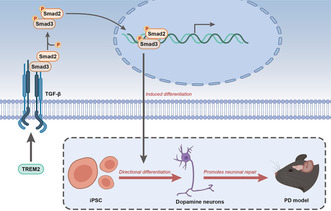

## 1 INTRODUCTION

Parkinson's disease (PD) is a fast‐growing neurodegenerative disorder,^1^, ^2^ showing easily‐recognized clinical syndromes and associating with a wide array of causes and clinical manifestations.^3^ Notably, PD is characterized by loss of dopamine neurons in the substantia nigra,^4^ and the majority of theratpeutic options are aimed at preventing this loss or providing replacement or reconstruction for the lost neurons or damaged neuronal circuits.^5^, ^6^ Cell replacement therapy using induced pluripotent stem cells (iPSCs) constitutes a promising strategy for the treatment of neurologic diseases, including PD, because of their potential to provide a large number of midbrain dopaminergic neurons.^7^, ^8^, ^9^ Elucidation of the mechanism underlying the differentiation of iPSCs into dopaminergic neurons can enable the development of novel therapies against PD.

Triggering receptor expressed on myeloid cells 2 (TREM2) is known as a transmembrane receptor of the immunoglobulin superfamily and also a key signaling hub for many pathological signaling pathways which mediate immunity.^10^ Variants of TREM2 have been largely reported to be associated with a variety of neurodegenerative disorders, including PD.^11^, ^12^ A recent study has verified that inhibition of TREM2 contributes to the occurrence and development of PD.^13^ Meanwhile, published data have suggested the important neuroprotective effects of TREM2 against PD as overexpression of TREM2 in BV2 microglia can promote M2 polarization and inhibit M1 microglial inflammatory responses and consequently protects dopaminergic neurons.^14^, ^15^ Besides, TREM2 is capable of modulating Aβ accumulation in a 5XFAD mouse model of Alzheimer's disease, thereby reducing neuronal damage.^16^

Bioinformatics analysis of the current study revealed that TREM2 may activate transforming growth factor beta (TGF‐β) pathway in iPSC‐derived dopaminergic neurons. TGF‐β is a member of the TGF‐β superfamily that is encoded by 33 genes in mammals, composed of homo‐ and heterodimers involved in the control of cell proliferation, differentiation, wound healing and immune system.^17^ The TGF‐β pathway has been involved in the regulation of neuronal development and survival.^18^ Inactivation of the astrocytic TGF‐β pathway contributes to immune cell infiltration, inflammatory response and the resultant neuronal injury.^19^ Therefore, in the present study, we carried out to characterize the effect of TREM2 on the neuronal repair in mice with experimental PD and attempted to dissect out the underlying mechanism associated with mediation of the TGF‐β pathway.

## 2 MATERIALS AND METHODS

### 2.1 In silico prediction

The GeneCards and PHGKB databases were used to predict PD‐related targets with “Parkinson's Disease” as the keyword. For the prediction results of the GeneCards database, the top 100 genes were selected according to the Relevance score. PD‐related dataset GSE89562 (3 brain tissue samples from control mice and 3 brain tissue samples from PD mouse models) was downloaded from the GEO database.

Differential gene expression analysis was performed using the “limma” package in R. Genes were selected as differentially expressed if their |log2 fold change (FC)| > 0.5 and adj. *p*‐value < 0.01. Furthermore, GSEA was adopted to reveal the difference of enriched pathways between induced pluripotent stem cell (iPSC)‐derived neurons from two healthy controls and two TREM2 mutant patients in the GSE143951 dataset.

### 2.2 Cell culture and grouping

iPSCs (Catalog No: EY‐Y9871, Shanghai YiYan Biotechnology Co., Ltd.) were seeded at a density of 5 × 10^5^ cells/mL in 6‐well plates and culture flasks pre‐coated with poly‐L‐lysine (PLL). They were induced for differentiation using DMEM/F12 medium supplemented with 10% fetal bovine serum (FBS), 2% B27 supplement, 2 mmol/L L‐glutamine, 100 U/mL penicillin, and 100 U/mL streptomycin. The experimental design consisted of six groups: iPSC + oe‐NC, iPSC + oe‐TREM2, iPSC + oe‐TGF‐β1, iPSC + oe‐NC + sh‐NC, iPSC + oe‐TREM2 + sh‐NC, and iPSC + oe‐TREM2 + sh‐TGF‐β1. Transfection was performed using the Lipofectamine 2000 reagent kit (Invitrogen, Carlsbad, CA, USA). A mixture of 4 μg plasmid DNA and 10 μL Lipofectamine 2000 diluted in 250 μL serum‐free Opti‐MEM medium (Gibco) was prepared and gently mixed. After incubating at room temperature for 5 min, the mixture was added to the culture wells and incubated at 37°C, 5% CO_2_ in a cell culture incubator. After 6 h, the medium was replaced with complete culture medium and the cells were further incubated for 48 h before collection for assessing transfection efficiency and subsequent experiments.

### 2.3 Establishment of a mouse model of PD

Animal experiments were approved by the Animal Ethics Committee of Sichuan Provincial People's Hospital. Thirty‐six C57BL/6 mice (aged 3–6 weeks, weighing 25–30 g; SJA Laboratory Animal Co., Ltd.) were selected for this study. The PD mouse model was established by stereotaxic injection of 6‐hydroxydopamine (6‐OHDA) into the right striatum. Mice were stereotaxically injected with 1 μL of 6‐OHDA (3.4 μg/μL; containing 0.1% ascorbic acid; Sigma). The mice in the sham group were injected with equal volume of normal saline containing 0.1% ascorbic acid (*n* = 6).^20^ Three weeks later, mice underwent behavioral tests to verify the success of model construction. Cylinder test: mice were placed in a transparent plastic cylinder (10 × 20 cm), followed by counting the number of mice standing with their hind legs after 5 min. Rotation test: mice were injected intraperitoneally with 0.5 mg/kg of APO. After 5 min, rotation data were recorded for 30 min, and mice at 7 laps per minute were considered a successful model.^21^

The 6‐OHDA‐lesioned mice were stereotaxically injected with 2 μL normal saline (PD + NS), 2 μL untreated iPSC suspension (1 × 10^6^ cells/mL; PD + iPSC), 2 μL iPSC suspension transfected with oe‐NC (1 × 10^6^ cells/mL; iPSC + oe‐NC), and 2 μL iPSC suspension transfected with oe‐TREM2 (1 × 10^6^ cells/mL; iPSC + oe‐TREM2) (*n* = 6 mice for each group).

### 2.4 HE staining

Nigral tissues were dehydrated, embedded in paraffin and sectioned into three small sections. The sections were then dewaxed with xylene (I, II) for 10 min, rehydrated in descending series of alcohol for 5 min and stained with Mayer's hematoxylin for 4 min. Next, the sections were blued in ammonia water and dehydrated in ascending series of alcohol, and counterstained with eosin for 5 min. After blocking with neutral balsam, the sections were observed under a light microscope (CX41, Olympus Optical Co., Ltd, Tokyo, Japan).

### 2.5 Immunohistochemical staining

Nigral tissue sections were subjected to microwave‐stimulated antigen retrieval with 1 mM Tris‐EDTA (pH 8.0) and treated with 3% H_2_O_2_ at room temperature for 10 min. Next, sections were immunostained with primary antibodies to TH (1:500, ab137869, Abcam Inc., Cambridge, UK) and DAT (1:500, ab128848, Abcam) at 4°C overnight. The next day, the sections were incubated with polymer enhancer (PV‐9000, ZSGB‐Bio, Beijing, China) at room temperature for 20 min, and then with enzyme‐labeled anti‐mouse/rabbit polymer (PV‐9000, ZSGB‐Bio) at room temperature for 30 min. Thereafter, the sections were developed with DAB for 5 min, counterstained with hematoxylin, hydrolyzed, blued, dehydrated, cleared and mounted. Finally, the sections were observed under an inverted microscope (CX41, Olympus).

### 2.6 qRT‐PCR

Total RNA was extracted from nigral tissues using TRIzol reagent (Invitrogen), concentration and purity of which were measured with a NanoDrop 2000 UV spectrophotometer (1011 U, NanoDrop Technologies Inc.). RNA was then reverse transcribed to generate cDNA using PrimeScript RT reagent Kit (RR047A, Takara, Japan). Primers for PON1, LRRK2, HEXA, NR4A2, POLG, BDNF, TREM2, SNCA and MAPT were designed and synthesized by TaKaRa (Table S1). qRT‐PCR was performed using a ABI 7500 quantitative PCR instrument (Applied Biosystems, Foster City, CA). GAPDH was used as an internal reference and the fold changes were calculated using $2^{-∆∆C_{T}}$.

### 2.7 Western blot

Total protein was extracted from tissues and cells using RIPA lysis buffer (R0010, Beijing Solarbio Science & Technology Co., Ltd., Beijing, China) supplemented with 1% phosphorylase inhibitor and 1% protease inhibitor, with the concentration determined using a BCA protein assay kit (GBCBIO Technologies Inc., Guangzhou, China). Next, the protein was separated with 10% SDS‐PAGE and transferred onto PVDF membranes (Millipore, Billerica, MA). The membrane was blocked with TBST with 5% BSA at room temperature and probed overnight at 4°C with primary rabbit antibodies to TREM2 (1:1000, #61788, Cell Signaling Technologies, Beverly, MA), TGF‐β1 (1:800, ab215715, Abcam), Smad2/3 (1:500, ab236030, Abcam), p‐Smad2/3 (1:1500, ab272332, Abcam), DAT (1:200, ab128848, Abcam), TH (1:5000, ab137869, Abcam), and GAPDH (ab9485, 1:2500, Abcam) and then with secondary antibody goat anti‐rabbit IgG (1:10,000, ab97051, Abcam) at room temperature. ECL reagent was used to visualize the results by Image Quant LAS 4000C Gel Imager (GE Healthcare, Waukesha, WI). ImageJ software (National Institutes of Health) was applied for band intensity quantification, with GAPDH as an internal reference.

### 2.8 Immunofluorescence staining

Paraffin‐embedded nigral tissues were cut into 6‐μm‐thick sections using a microtome (CM1950; Leica, Heerbrugg, Switzerland), antigen retrieved in sodium citrate buffer (100×, pH 6.0) for 5 min, and incubated with 5% goat serum for 1 h to prevent nonspecific binding. Sections were then probed with primary antibodies to TH (1:250, ab137869, Abcam) for 4°C overnight and then with secondary antibody goat anti‐rabbit IgG (Alexa Flug 488) (1:1000, ab150077, Abcam) for 1 h at room temperature. Thereafter, the sections were stained with DAPI for 5 min in the dark and observed under a confocal laser microscope (Olympus).

### 2.9 Statistical analysis

Measurement data are presented as mean ± standard deviation. All statistical analyses in this study were performed using SPSS 19.0 software (IBM Corp. Armonk, NY). The normality of the distribution was tested using the Kolmogorov–Smirnov test. Paired *t*‐test or unpaired Student's *t*‐test was used to compare two groups with normally distributed data. One‐way ANOVA was used for comparing data among multiple groups, followed by Tukey's post hoc test. For data that did not follow a normal distribution, non‐parametric tests were employed. A *p* value < 0.05 was considered significant.

## 3 RESULTS

### 3.1 TREM2 is poorly expressed in the nigral tissues of PD mouse models

**FIGURE 1:**
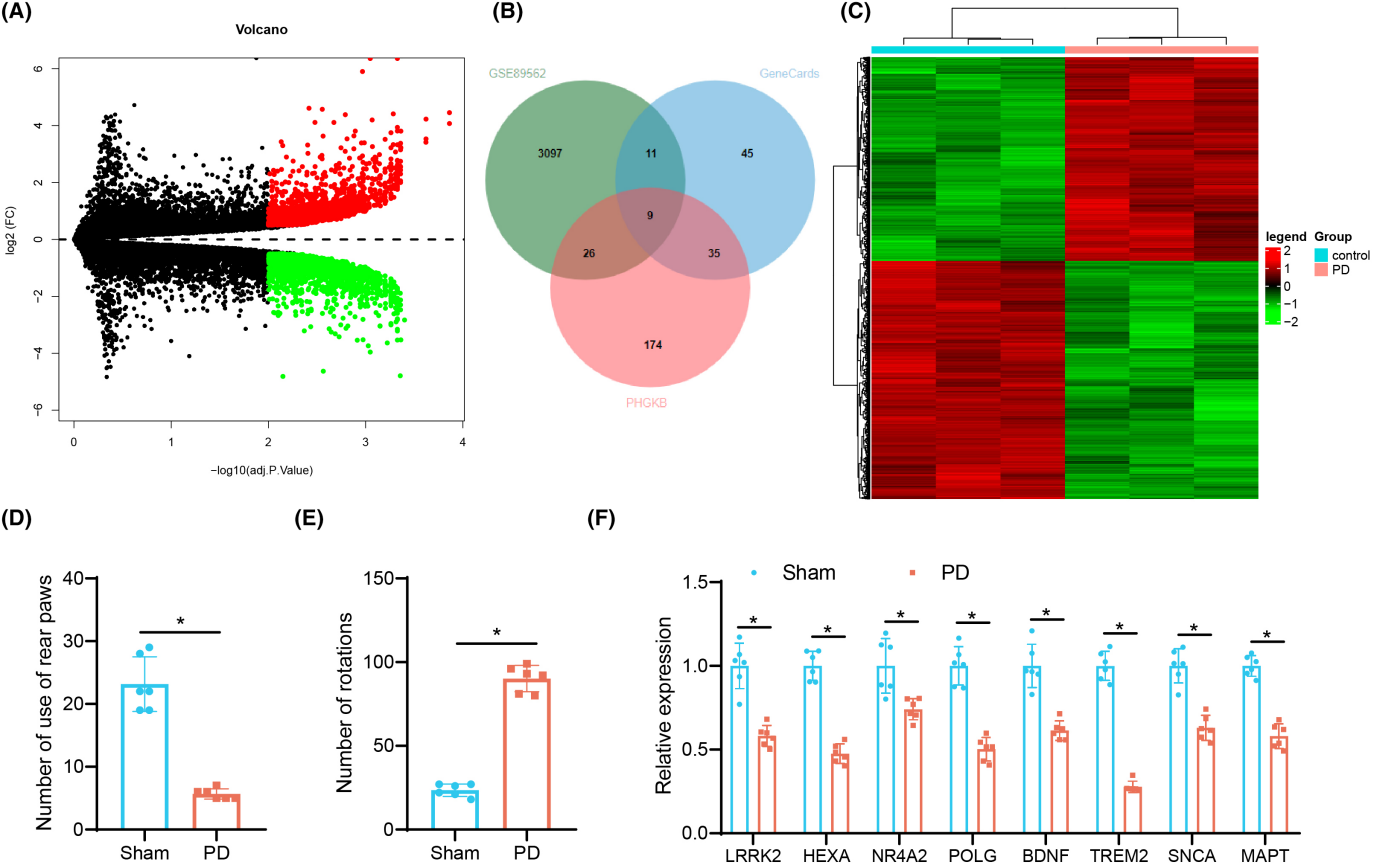
Differentially expressed genes in the nigral tissues of PD mouse models. (A) A volcano map of the differentially expressed genes between three brain tissue samples from control mice and three brain tissue samples from PD mouse models in the GSE89562 dataset. Red indicates upregulated genes and green indicates downregulated genes. (B) Venn diagram of the PD‐related genes predicted by GeneCards and PHGKB databases and the differentially expressed genes in PD samples in the GSE89562 dataset. (C) A heat map of the differentially expressed genes between three brain tissue samples from control mice and three brain tissue samples from PD mouse models in the GSE89562 dataset. Nine PD‐related genes are marked in the figure. (D) Motor function of sham‐operated and 6‐OHDA‐lesioned mice determined by cylinder test. (E) Motor function of sham‐operated and 6‐OHDA‐lesioned mice determined by rotation test. (F) The expression of differentially expressed genes in the nigral tissues of sham‐operated and 6‐OHDA‐lesioned mice determined by qRT‐PCR. *p < 0.05. *n* = 6 mice for each treatment.

First, we conducted differential analysis of the PD‐related dataset GSE89562 to determine the key genes involved in the progression of PD. The results yielded 3143 differentially expressed genes, including 1448 upregulated and 1695 downregulated genes (Figure 1A). Next, these genes were intersected with the PD‐related targets predicted by GeneCards and the PHGKB databases, and nine genes were obtained (Figure 1B). As shown in Figure 1C, PON1 was an upregulated gene among the nine PD‐related genes while LRRK2, HEXA, NR4A2, POLG, BDNF, TREM2, SNCA and MAPT were downregulated genes.

Then, to explore the potential therapeutic targets of PD, a PD mouse model induced by 6‐OHDA was constructed. The results of cylinder and rotation tests suggested a decline in the number of mice standing with hind legs and the number of rotations in the 6‐OHDA‐lesioned mice (Figure 1D,E), which showed the impaired motor function and the successful establishment of the PD mouse model. In addition, qRT‐PCR data revealed that TREM2 exhibited the lowest expression in the nigral tissues of 6‐OHDA‐lesioned mice among the nine differentially expressed genes (Figure 1F).

Therefore, we suspected that TREM2 can be used as a potential therapeutic target for PD.

### 3.2 Overexpression of TREM2 promotes the directed differentiation of iPSCs into dopaminergic neurons

**FIGURE 2:**
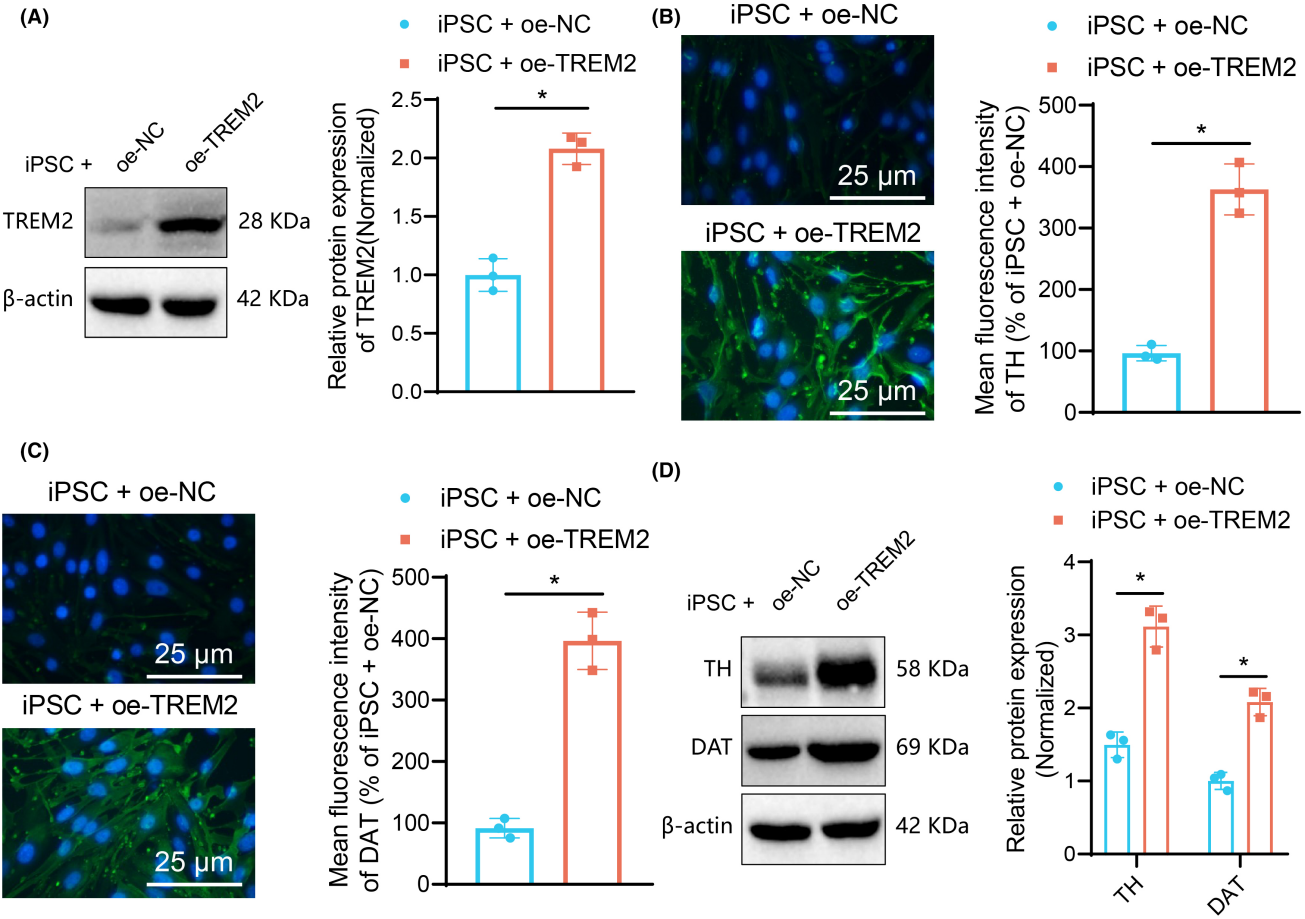
TREM2 enhances the directed differentiation of iPSCs into dopaminergic neurons. (A) Western blot of TREM2 protein in iPSCs transfected with oe‐TREM2. (B) Immunofluorescence staining of TH protein in iPSCs transfected with oe‐TREM2 (scale bar: 25 μm). (C) Immunofluorescence staining of DAT protein in iPSCs transfected with oe‐TREM2. (D) Western blot of TH and DAT proteins in iPSCs transfected with oe‐TREM2. *p < 0.05. Cell experiments were repeated independently three times.

Recent data have shown that iPSC‐derived dopaminergic neurons are an expected source for cell‐based therapies for PD.^22^ Here, to examine whether TREM2 can promote the differentiation of iPSCs into dopaminergic neurons, we overexpressed TREM2 in iPSCs. Western blot results showed an increase in TREM2 expression in iPSCs transfected with oe‐TREM2 (Figure 2A), which proved the effective transfection. In addition, overexpression of TREM2 was found to elevate the expression of dopaminergic neuron markers TH and DAT (Figure 2B–D). The above results demonstrated that overexpression of TREM2 can enhance the directed differentiation of iPSCs into dopaminergic neurons.

### 3.3 TREM2 activates the TGF‐β pathway

**FIGURE 3:**
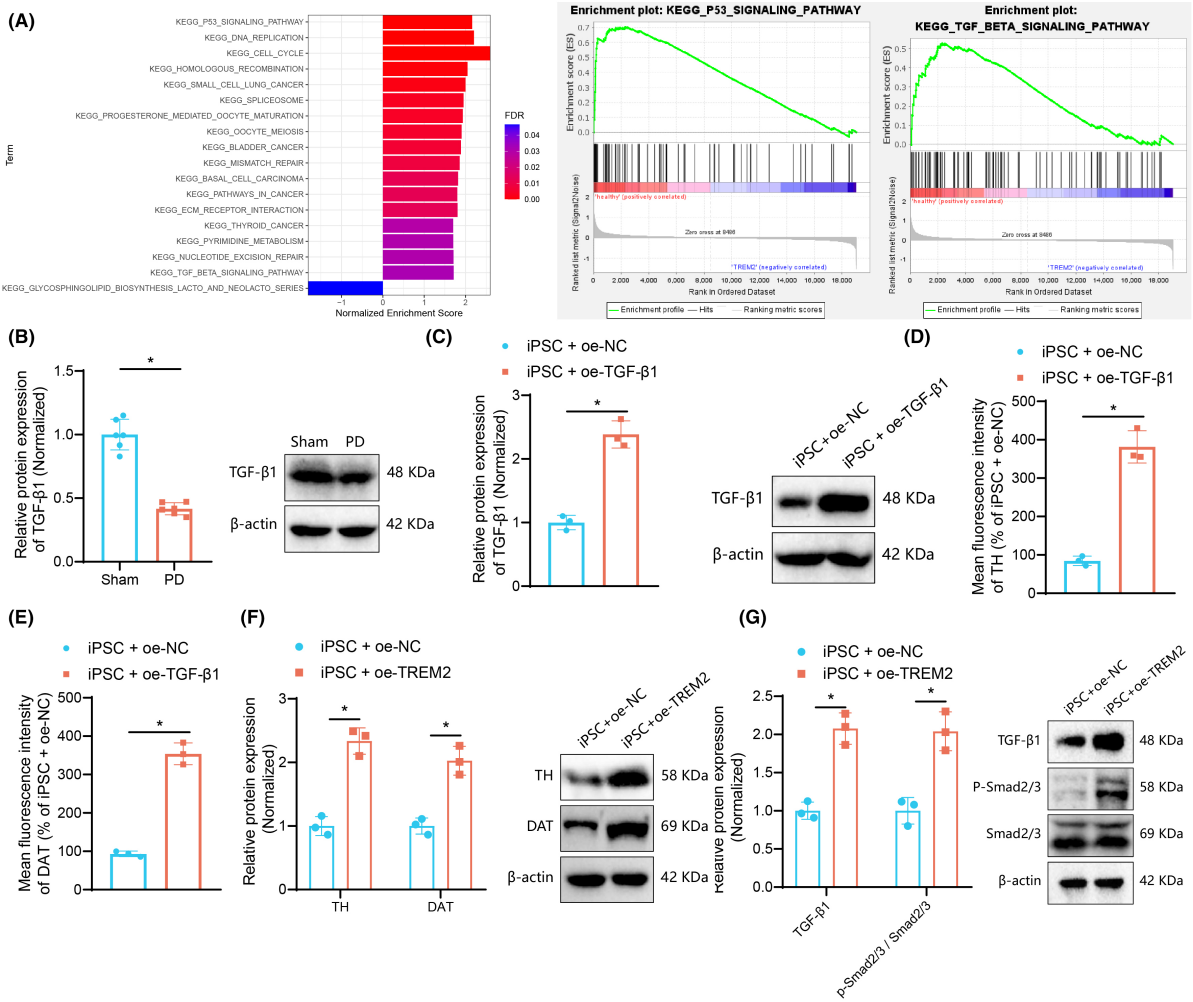
TREM2 facilitates activation of the TGF‐β1 pathway. (A) GSEA analysis of the difference of enriched pathways between iPSC‐derived neurons from two healthy controls and two TREM2 mutant patients in the GSE143951 dataset. (B) Western blot of TGF‐β1 protein in the nigral tissues of sham‐operated and 6‐OHDA‐lesioned mice. (C) Western blot of TGF‐β1 protein in iPSCs transfected with oe‐TGF‐β1. (D) Immunofluorescence staining of TH protein in iPSCs transfected with oe‐TGF‐β1. (E) Immunofluorescence staining of DAT protein in iPSCs transfected with oe‐TGF‐β1. (F) Western blot of TH and DAT proteins in iPSCs transfected with oe‐TGF‐β1. (G) Western blot of TGF‐β1 pathway‐related proteins in iPSCs transfected with oe‐TREM2. *p < 0.05. *n* = 6 mice for each treatment. Cell experiments were repeated independently three times.

To elucidate the mechanistic role of TREM2 in iPSC‐derived dopaminergic neurons, we performed GSEA analysis on the iPSC‐derived neuron dataset GSE143951, which included samples from two healthy controls and two TREM2 mutation carriers. The results showed a significant decrease in enrichment of the “P53_SIGNALING_PATHWAY” and “TGF_BETA_SIGNALING_PATHWAY” following TREM2 mutation (Figure 3A). The p53 signaling pathway has been implicated as an initiating factor in dopamine cell apoptosis,^23^ whereas TGF‐β is known to enhance differentiation, survival, and maintenance of pluripotent stem cell‐derived dopaminergic neurons.^24^ Therefore, we hypothesize that TREM2 may activate the TGF‐β signaling pathway in iPSC‐derived dopaminergic neurons.

Moreover, western blot results exhibited that TGF‐1β protein expression was downregulated in the nigral tissues of 6‐OHDA‐lesioned mice (Figure 3B). Additionally, western blot confirmed the transfection efficiency of oe‐TGF‐β1 in iPSCs, as shown by upregulated TGF‐1β protein expression in iPSCs transfected with oe‐TGF‐β1 (Figure 3C).

Immunofluorescence and western blot results showed an enhancement in the protein expression of TH and DAT in iPSCs transfected with oe‐TGF‐β1 (Figure 3D–F, Figure S1A,B). This indicated that TGF‐β1 could promote the directed differentiation of iPSCs into dopaminergic neurons. Further, western blot results demonstrated that TREM2 overexpression increased TGF‐β1 protein expression and Smad2/3 phosphorylation levels (Figure 3G).

Altogether, TREM2 could activate the TGF‐β1 pathway.

### 3.4 TREM2 promotes the directed differentiation of iPSCs into dopaminergic neurons by activating the TGF‐β1 pathway

**FIGURE 4:**
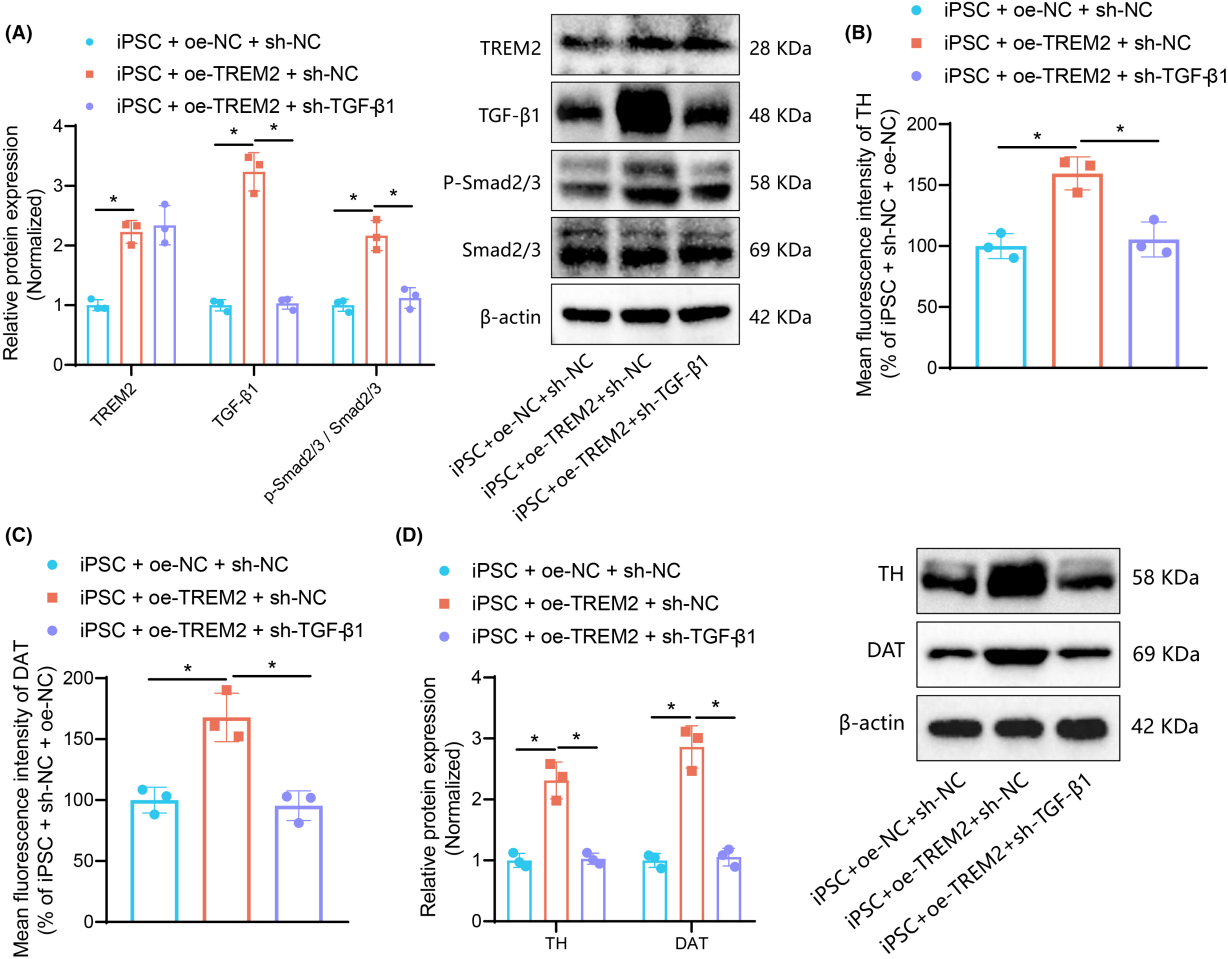
TREM2 increases the directed differentiation of iPSCs into dopaminergic neurons by activating the TGF‐β1 pathway. **(**A) Western blot of TREM2, TGF‐β1 and Smad2/3 proteins in iPSCs transfected with sh‐TREM2 or combined with oe‐TGF‐β1. (B) Immunofluorescence staining of TH protein in iPSCs transfected with sh‐TREM2 or combined with oe‐TGF‐β1. (C) Immunofluorescence staining of DAT protein in iPSCs transfected with sh‐TREM2 or combined with oe‐TGF‐β1. (D) Western blot of TH and DAT proteins in iPSCs transfected with sh‐TREM2 or combined with oe‐TGF‐β1. *p < 0.05. Cell experiments were repeated independently three times.

To validate this hypothesis, we conducted a grouping analysis of iPSCs. Western blot results (Figure 4A) revealed that compared to the iPSC + oe‐NC + sh‐NC group, the iPSC + oe‐TREM2 + sh‐NC group exhibited significantly increased expression levels of TREM2 and TGF‐β1 proteins, as well as phosphorylation levels of Smad2/3. Furthermore, compared to the iPSC + oe‐TREM2 + sh‐NC group, the iPSC + oe‐TREM2 + sh‐TGF‐β1 group showed significant decreases in the expression levels of TGF‐β1 protein and phosphorylation levels of Smad2/3, whereas TREM2 exhibited no significant changes. Immunofluorescence and western blot results (Figure 4B–D, Figure S1C,D) demonstrated that compared to the iPSC + oe‐NC + sh‐NC group, the iPSC + oe‐TREM2 + sh‐NC group displayed a significant increase in the expression levels of TH and DAT proteins. Additionally, compared to the iPSC + oe‐TREM2 + sh‐NC group, the iPSC + oe‐TREM2 + sh‐TGF‐β1 group exhibited a significant decrease in the expression levels of TH and DAT proteins.

Collectively, these findings suggest that TREM2 can promote the directed differentiation of iPSCs into dopaminergic neurons through the activation of the TGF‐β signaling pathway.

### 3.5 iPSCs overexpressing TREM2 enhance neuronal repair in 6‐OHDA‐lesioned mice

**FIGURE 5:**
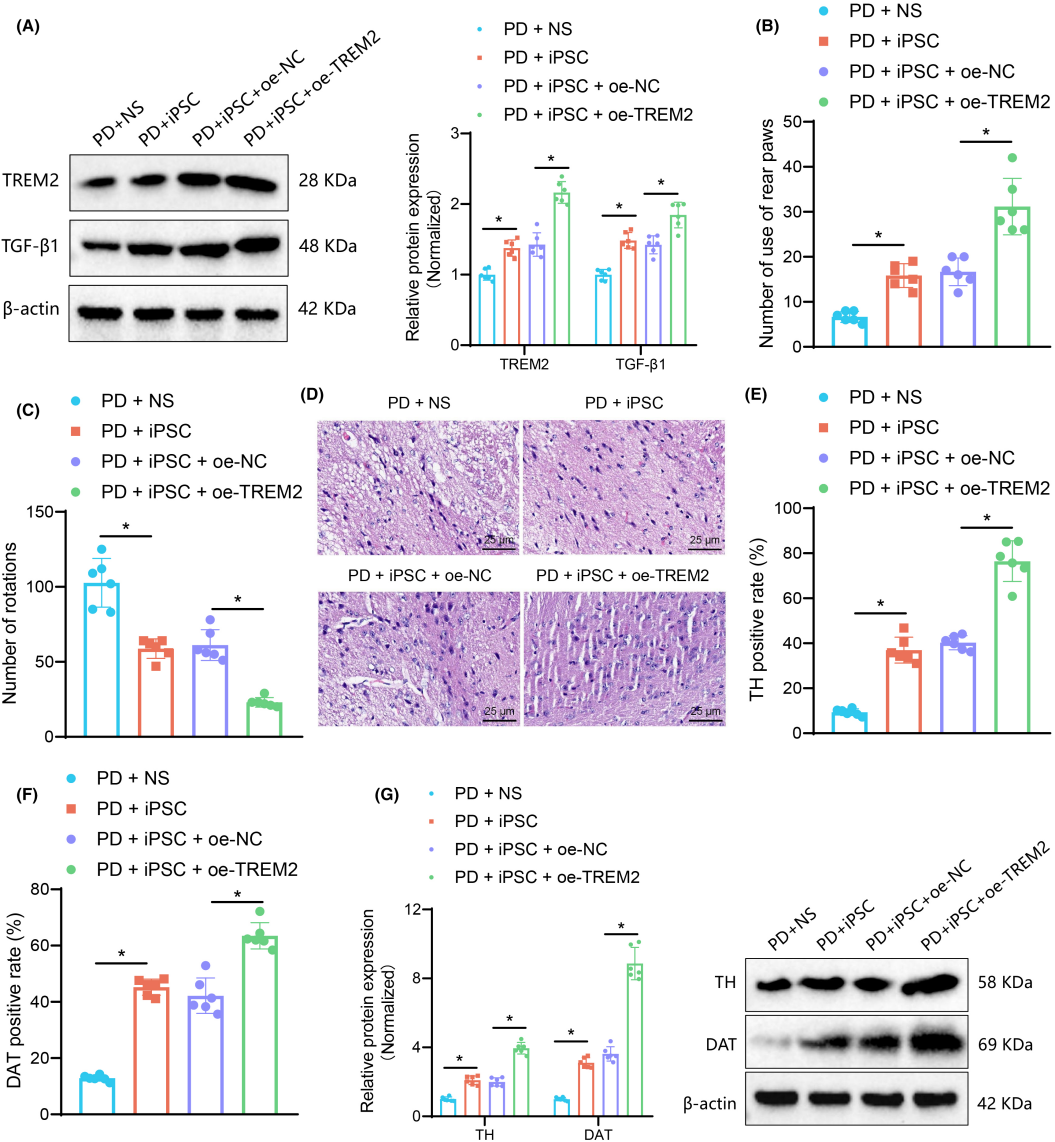
TREM2 overexpressing iPSCs alleviate dopaminergic neuron injury in 6‐OHDA‐lesioned mice. (A) Western blot of TREM2 and TGF‐β1 proteins in the nigral tissues of 6‐OHDA‐lesioned mice treated with iPSC suspension or combined with oe‐TREM2. (B) Motor function of 6‐OHDA‐lesioned mice treated with iPSC suspension or combined with oe‐TREM2 determined by cylinder test. (C) Motor function of 6‐OHDA‐lesioned mice treated with iPSC suspension or combined with oe‐TREM2 determined by rotation test. (D) HE staining of nigral tissues of 6‐OHDA‐lesioned mice treated with iPSC suspension or combined with oe‐TREM2 (scale bar: 25 μm). (E) Immunohistochemistry of TH protein in nigral tissues of 6‐OHDA‐lesioned mice treated with iPSC suspension or combined with oe‐TREM2. (F) Immunohistochemistry of DAT protein in nigral tissues of 6‐OHDA‐lesioned mice treated with iPSC suspension or combined with oe‐TREM2. (G) Western blot of TH and DAT proteins in nigral tissues of 6‐OHDA‐lesioned mice treated with iPSC suspension or combined with oe‐TREM2. *p < 0.05. *n* = 6 mice for each treatment.

Finally, we sought to verify whether TREM2 plays a role in neuronal repair in 6‐OHDA‐lesioned mice. Western blot results presented that the protein expression of TREM2 and TGF‐β1 was increased in the nigral tissues of 6‐OHDA‐lesioned mice injected with iPSC suspension and a more pronounced increase was noted in the presence of TREM2 overexpression (Figure 5A).

Moreover, behavioral test results suggested that the motor function of 6‐OHDA‐lesioned mice injected with iPSC suspension was improved, which was more evident following TREM2 overexpression (Figure 5B,C). These data implied that iPSCs overexpressing TREM2 could improve the behavioral characteristics of 6‐OHDA‐lesioned mice.

In addition, HE staining results showed that neurons in nigral tissues of 6‐OHDA‐lesioned mice injected with saline were obviously distorted, angulated, and atrophied, with vacuoles in the intercellular space, and different signs of degeneration and necrosis, showing a deep eosinophilic cytoplasm, accompanied by pyknosis and nucleolysis. These changes were remarkably ameliorated as shown by the presence of multipolar neurons with nucleoli and basophilic granular cytoplasm in the substantia nigra pars compacta. This presence was more significant following TREM2 overexpression (Figure 5D).

Immunohistochemistry and western blot results identified increased TH and DAT expression in the nigral tissues of 6‐OHDA‐lesioned mice injected with iPSC suspension and this increase was more considerable in response to TREM2 overexpression (Figure 5E–G, Figure S1E,F).

In summary, dopaminergic neuron injury was improved in 6‐OHDA‐lesioned mice after TREM2 overexpression in iPSCs.

## 4 DISCUSSION

TREM2 confers neuroprotective functions against neuronal injury in a variety of neurodegenerative diseases.^25^, ^26^ This study investigated the mechanism of TREM2 affecting the neuronal repair in 6‐OHDA‐induced mouse models of PD and demonstrated that TREM2 overexpression can potentially promote the directed differentiation of iPSCs into dopaminergic neurons by activating the TGF‐β1 pathway, thereby enhancing neuronal repair in 6‐OHDA‐lesioned mice.

The results from this study first identified poorly expressed TREM2 in the nigral tissues of 6‐OHDA‐induced PD mouse models. In partially agreement with this finding, recent literature has revealed that both the mRNA and protein expression of TREM2 is downregulated in BV2 cells exposed to 6‐OHDA.^27^ Subsequent results of this study suggested that overexpression of TREM2 could facilitate the directed differentiation of iPSCs into dopaminergic neurons. A recent study has identified iPSC‐derived dopaminergic neurons to be an expected source for the cell‐based therapies against PD.^22^ TH and DAT are well‐known markers of dopaminergic neurons and their densities serve as a biomarker to detect the integrity of dopaminergic neurons.^28^, ^29^, ^30^ In a previous study, 1‐methyl‐4‐phenyl‐1, 2, 3, 6‐tetrahydropyridine (MPTP)‐induced loss of TH‐positive neurons in the substantia nigra and the striatum is found to be markedly attenuated by TREM2 overexpression; moreover, elevated TH protein expression is detected in the TREM2‐overexpressed MPTP mice.^15^ These data highlight the involvement of TREM2 in PD and its role as a potential target for PD.

Mechanistic investigations in this study indicated that TREM2 activated the TGF‐β pathway and thus promoted the directed differentiation of iPSCs into dopaminergic neurons. This is the first study to report the relationship between TREM2 and the TGF‐β pathway and thus warrants further investigation for validation in the next study. An increasing number of growth factors, especially TGF‐β, have been implicated in the regulation of the differentiation of dopaminergic neurons.^31^, ^32^ TGF‐β, acting as a survival‐promoting factor, is capable of enhancing the differentiation, survival and maintenance of dopaminergic neurons derived from embryonic stem cells.^24^ In addition, treatment of ventral midbrain dissociated neurospheres with TGF‐β leads to increased number of TH‐immunoreactive cells and TGF‐β is essential for the differentiation of midbrain progenitors into dopaminergic neurons.^33^ These findings advance our understanding of the role of TREM2 regulating TGF‐β in the pathogenesis of PD, providing new insights into TREM2 functions in this disease.

Additionally, the current study demonstrated that iPSCs overexpressing TREM2 could enhance neuronal repair in 6‐OHDA‐lesioned mice. PD is a type of neurodegenerative disease, associated with degeneration of dopaminergic neurons.^28^ Neurodegeneration is a debilitating condition which can result in nerve cell degeneration or death.^34^ Overexpression of TREM2 has been reported to reduce dopaminergic neurodegeneration in the MPTP‐induced mouse model of PD.^11^ Additionally, knockout of TREM2 exacerbates dopaminergic neuron loss in a mouse model of PD induced by adeno‐associated viral vectors expressing human α‐syn.^27^ Meanwhile, human PSC‐derived neural cells can be used to replace degenerating cells, providing a promising cell‐based medicine for the recovery of brain function.^35^ Notably, reversing the heatstroke‐induced decrease in the expression of TGF‐β can reduce the neuroinflammation and nerve damage caused by heatstroke.^36^ Considering the aforementioned findings and evidence, TREM2 can serve as a beneficial role in functional neurological recovery, which is associated with its promoting effect on the TGF‐β pathway activation.

**FIGURE 6:**
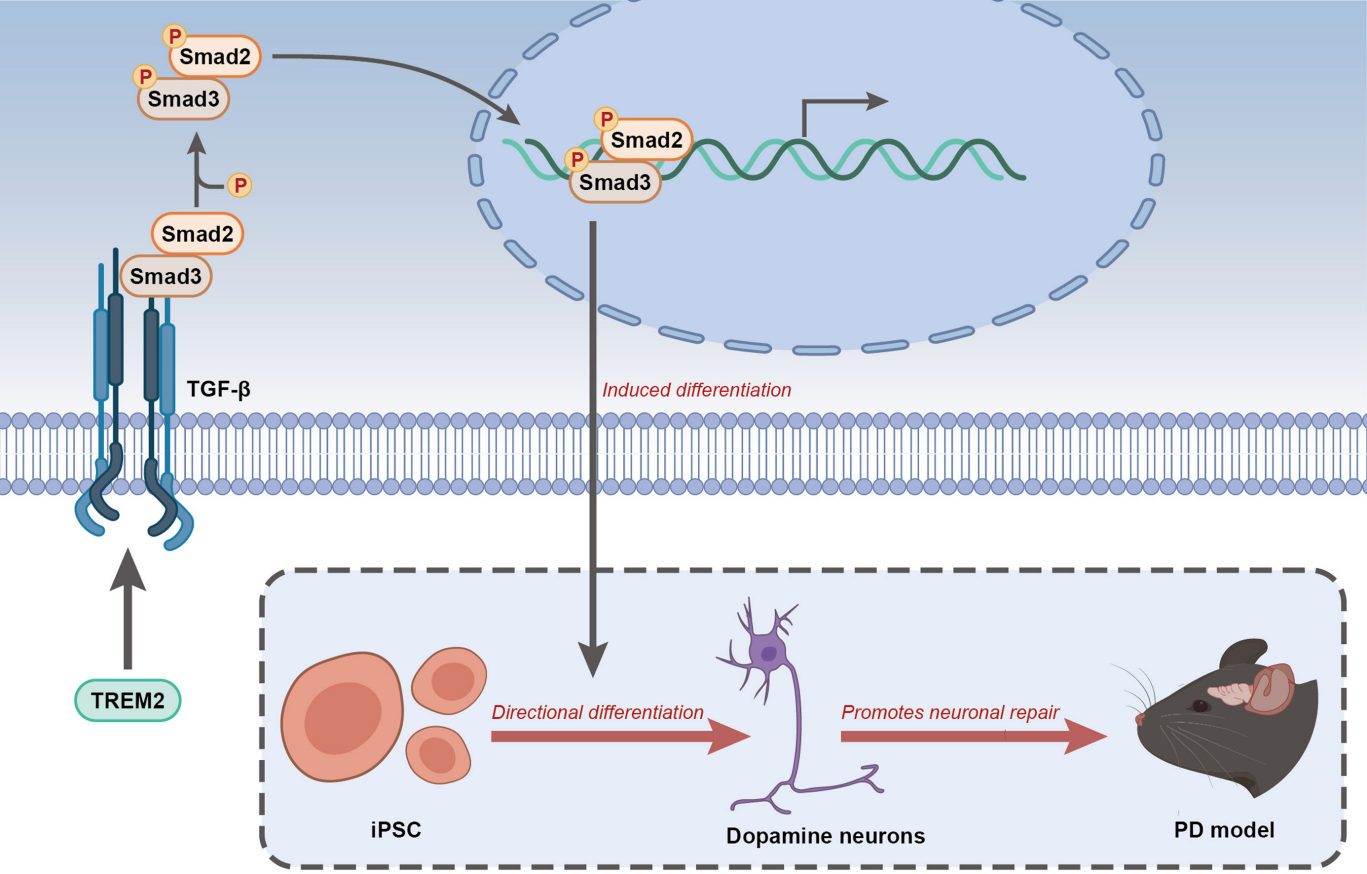
A molecular mechanism graph illustrating the involvement of TREM2 in the effect of iPSC differentiation in dopaminergic neuron injury. TREM2 may induce directed differentiation of iPSCs into dopaminergic neurons through activation of the TGF‐β1 pathway and thus promotes neuronal repair in PD.

In summary, in the present study, we provide novel evidence that overexpression of TREM2 promotes neuronal repair in 6‐OHDA‐lesioned mice via modulation of differentiation of iPSCs into dopaminergic neurons through activation of the TGF‐β pathway (Figure 6). These findings have opened new avenues for therapeutic intervention to delay or prevent the neuronal injury induced by neurodegenerative diseases.

## AUTHOR CONTRIBUTIONS

Hanbai Liang, Dongdong Zhao and Chunhui Wu conceived and designed research; Zijing Wang analyzed data; Hanbai Liang, Ping Liu, Cheng Yin and Huan Xiong performed experiments, interpreted results of experiments, prepared figures; and all authors contributed to drafting, editing and revising this manuscript. All authors read and approved final version of manuscript.

## FUNDING INFORMATION

The present study was supported by the Fundamental Research Funds for the Central Universities (ZYGX2021YGLH010) and Key Research & Development program of the Science and Technology Department of Sichuan Province (Social Development) (2022YFS0142); Sichuan Province cadres health research project (2023104).

## CONFLICT OF INTEREST STATEMENT

The authors declare that they have no competing interests.

## CONSENT FOR PUBLICATION

Not applicable.

## Supporting information


Figure S1.

Table S1.

## Data Availability

Research data are not shared.
