# Supplementary figures and images for: TREM2 gene induces differentiation of induced pluripotent stem cells into dopaminergic neurons and promotes neuronal repair via TGF‐β activation in 6‐OHDA‐lesioned mouse model of Parkinson's disease
Source: CNS Neurosci Ther. 2024 Feb 13;30(2):e14630. doi: 10.1111/cns.14630 (PMC10862187; doi:10.1111/cns.14630)

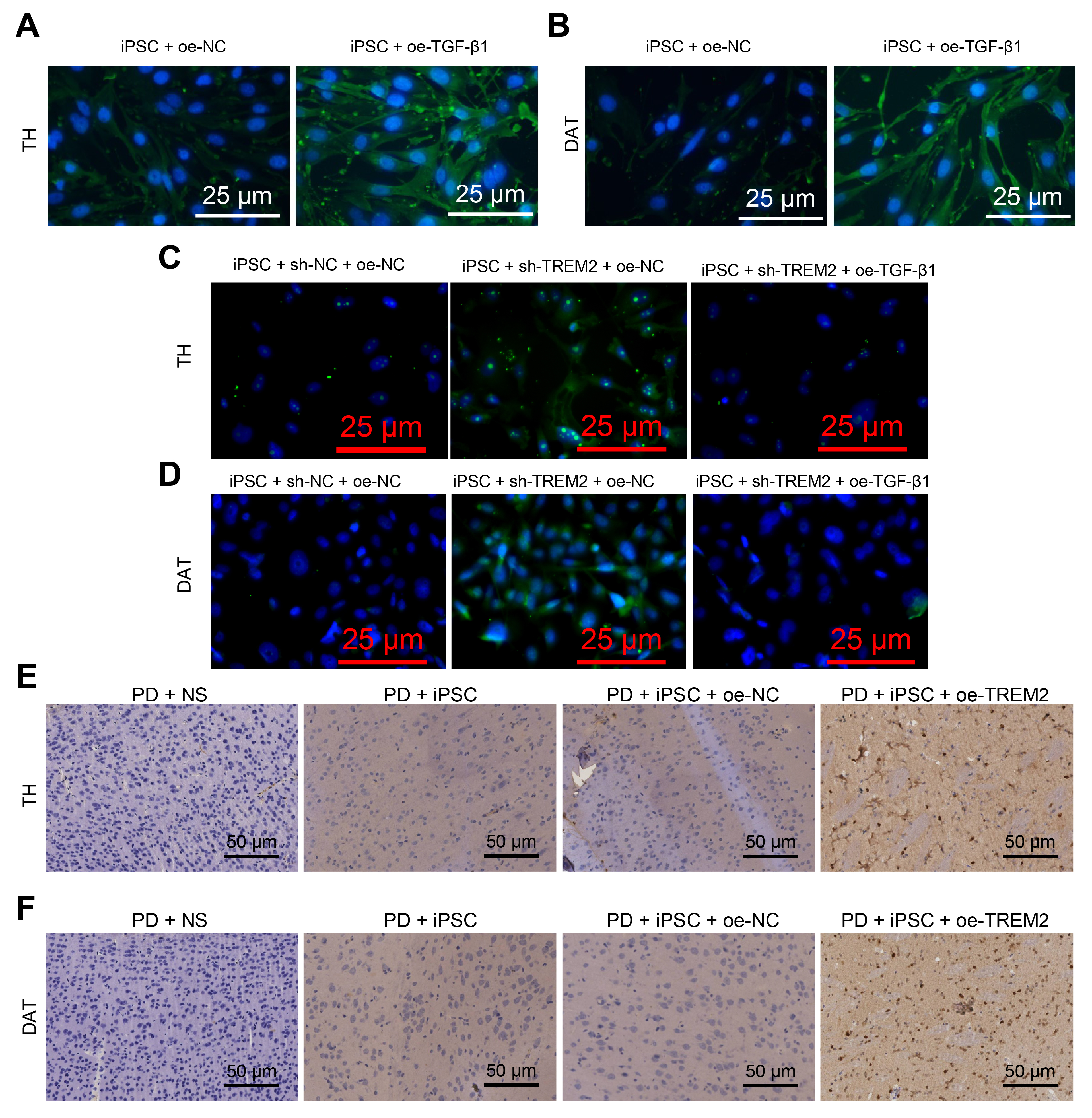

Supplement: Supplementary file 1 — Figure S1. [file CNS-30-e14630-s001.jpg]
