# Supplementary material for: TREM2 gene induces differentiation of induced pluripotent stem cells into dopaminergic neurons and promotes neuronal repair via TGF‐β activation in 6‐OHDA‐lesioned mouse model of Parkinson's disease
Source: CNS Neurosci Ther. 2024 Feb 13;30(2):e14630. doi: 10.1111/cns.14630 (PMC10862187; doi:10.1111/cns.14630)
Supplement: Supplementary file 2 — Table S1. [file CNS-30-e14630-s002.docx]

**Supplementary Table 1** Primer sequences of qRT-PCR

| Gene | Sequence (5'-3') |
| --- | --- |
| PON1 (mouse) | Forward: 5'-CCAGGTGGTAGCAGAAGGGT-3'  Reverse: 5'-CTGGATTCGAAGCACCTCTG-3' |
| LRRK2 (mouse) | Forward: 5'-GCCATGCACAGATATTCAGCC-3'  Reverse: 5'-CATGGCCTCCACCACAAGAT-3' |
| HEXA (mouse) | Forward: 5'-CTGCAGAATCCTTTGCTTACGG-3'  Reverse: 5'-CATGGTACCGGAACTGGAAGT-3' |
| NR4A2 (mouse) | Forward: 5'-GAGCTGGAGCTGGGCTG-3'  Reverse: 5'-TACTGCGCCTGAACACAAGG-3' |
| POLG (mouse) | Forward: 5'-GAGCGTTACCTGACAGAGGC-3'  Reverse: 5'-GGGCCAGGATCTTCTTTGCCTT-3' |
| BDNF (mouse) | Forward: 5'-CGGAGAGCAGAGTCCATTCAG-3'  Reverse: 5'-CCAGTATACCAACCCGGAGC-3' |
| TREM2 (mouse) | Forward: 5'-GTCACCTCTAGCCTACCACCT-3'  Reverse: 5'-GAAACTTGCTCAGGAGAACGC-3' |
| SNCA (mouse) | Forward: 5'-AGAGGAGCGAAGGCACGAG-3'  Reverse: 5'-CCTAGGCTTCTGAAGAACTCCG-3' |
| MAPT (mouse) | Forward: 5'-CGGAGACCTCCGATGCTAAG-3'  Reverse: 5'-GTCTCCGATGCCTGCTTCTT-3' |
| GAPDH (mouse) | Forward: 5'-ACCCTTAAGAGGGATGCTGC-3'  Reverse: 5'-ATCCGTTCACACCGACCTTC-3' |
